# Style

**Published:** 1879-07

**Authors:** 


**Style.**—Some men of vigorous minds, but more conversant with things than with words, and who, having never studied composition as an art, have not learned that real force of style must be effortless, and consists mainly in its simplicity and appropriateness ; they fancy that common words are not half strong enough to say what they want to say ; and so they try to strengthen them by writing them in a different character. Men of science do this ; for words with them are signs, which must stand out to be conspicuous. Soldiers often do this ; for, though a few of them are among the most skillful in the drilling and maneuvering of words, the chief part have no notion that a word may be louder than a cannon-ball, and sharper than a sword. Cobbett is profuse of italics. This instance may be supposed to refute the assertion, that the writers who use them are not versed in the art of composition. But, although Cobbett was a wonderful master of plain speech, all his writings betray his want of logical and literary culture. He had never sacrificed to the graces; who can not be won without many sacrifices. He cared only for strength ; and as his own bodily frame was of the Herculean, rather than the Apollonean cast, he thought that a man could not be very strong unless he displayed his thews. Besides, a Damascus blade would not have gashed his enemies enough for his taste; he liked to have a few notches on his sword.

